# COVID-19-induced Brugada phenocopy pattern in a patient with previous myocardial infarction: A case report

**DOI:** 10.1097/MD.0000000000046907

**Published:** 2025-12-26

**Authors:** Lijiang Ding, Huayong Jin

**Affiliations:** a Department of Electrocardiology, Shaoxing People’s Hospital, School of Medicine, Shaoxing University, Shaoxing, Zhejiang Province, China.

**Keywords:** Brugada pattern, COVID-19, electrocardiogram, old myocardial infarction

## Abstract

**Rationale:**

Coronavirus disease 2019 (COVID-19) can cause electrocardiographic changes, such as ST-segment elevation, by increasing the dispersion of cardiac repolarization or cause delayed depolarization in a localized area of the right ventricular outflow, leading to clinical misdiagnosis.

**Patient concerns:**

A 57-year-old man with a history of hypertension and previous myocardial infarction presented to the emergency department with a 12-hour history of fever, chest tightness, and palpitations.

**Diagnoses:**

An emergency electrocardiogram (ECG) showed sinus rhythm with abnormal Q waves and ST-segment elevation (0.1–0.35 mV) in leads V1–V3. A positive COVID-19 polymerase chain reaction test and specific ECG metrics, including β-angle measurement and r’-wave triangle base duration, led to the diagnosis of a Brugada phenocopy pattern triggered by COVID-19.

**Interventions:**

The patient was treated with an antiplatelet agent (clopidogrel), an antipyretic (acetaminophen) for fever, and oral antivirals (nimatrelvir/ritonavir).

**Outcomes:**

After 2 days, his temperature normalized, and a repeat ECG showed resolution of ST-segment elevation.

**Lessons:**

This case highlights the importance of considering fever-induced Brugada phenocopy in the differential diagnosis of ST-segment elevation in patients with coronary artery disease. Careful ECG analysis, including assessment of the β-angle and r’-wave morphology, is crucial to avoid misdiagnosis of ST-elevation myocardial infarction. Initial management should focus on controlling triggering factors such as fever with antipyretics and antivirals to prevent malignant arrhythmias.

## 1. Introduction

Common causes of recurrent ST-segment elevation in patients with a history of myocardial infarction include ventricular aneurysm, re-occlusion of the culprit artery, and acute pericarditis. Emerging evidence suggests that fever can induce electrocardiographic (ECG) changes resembling a Brugada pattern,^[1]^ a phenomenon known as Brugada phenocopy, in patients with previous anterior wall myocardial infarction, necessitating careful ECG analysis, including β-angle measurement and r’-wave triangle base duration, to avoid misdiagnosis.

## 2. Case presentation

**Figure 1.**
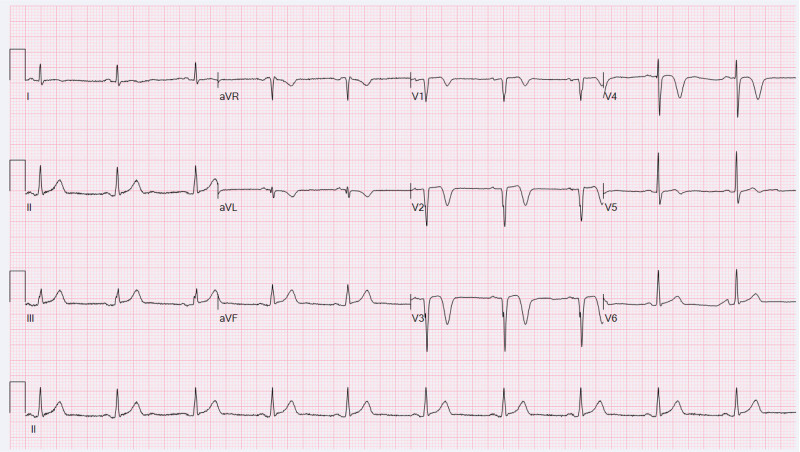
Electrocardiogram obtained after percutaneous coronary intervention 2 years prior to the current presentation.

**Figure 2.**
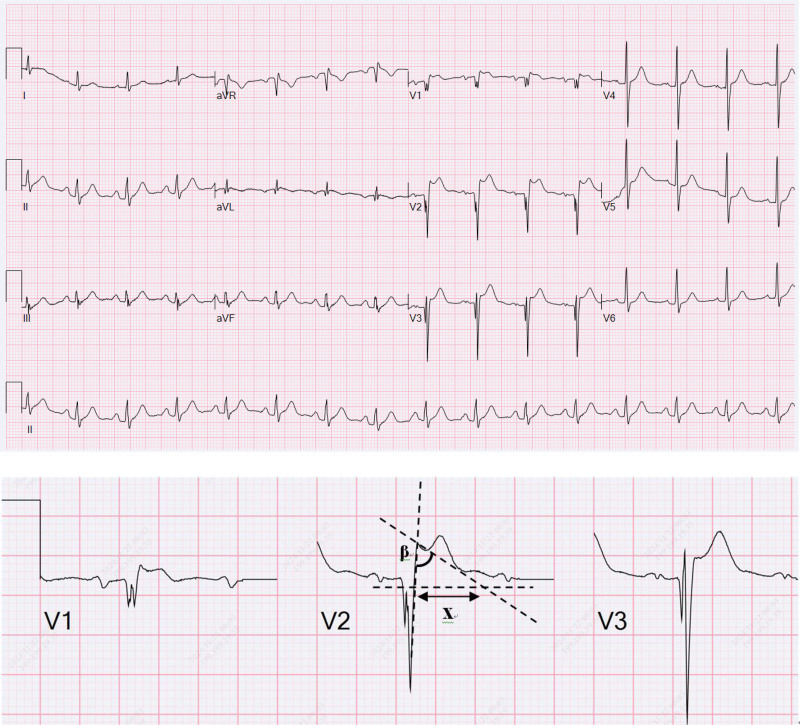
Emergency electrocardiogram of the patient. The β-angle is formed between the ascending slope of the S wave and the descending slope of the r’-wave. The variable × represents the duration of the base of the triangle located 5 mm from the peak of the r’-wave.

A 57-year-old man presented to the emergency department with a half-day history of fever, chest tightness, and palpitations. His medical history included hypertension and an acute anterior wall myocardial infarction 2 years earlier, for which he underwent emergency percutaneous coronary intervention with implantation of a drug-eluting stent in the proximal left anterior descending artery (Fig. 1). His regular medications included clopidogrel and a statin. On admission, his vital signs were as follows: temperature, 39.5°C; pulse, 95 bpm; and blood pressure, 123/80 mm Hg. Physical examination was unremarkable. An emergency ECG (Fig. 2) revealed sinus rhythm with abnormal Q waves and ST-segment elevation (0.1–0.35 mV) in leads V1–V3. Cardiac troponin and other myocardial enzyme levels were within normal limits. Echocardiography showed normal chamber size, an intact septal structure, normal myocardial thickness, and coordinated wall motion without segmental abnormalities at rest. No pericardial effusion was observed, and the left ventricular ejection fraction was 69%. Coronavirus disease 2019 (COVID-19) polymerase chain reaction test was positive.

## 3. ECG interpretation and clinical course

**Figure 3.**
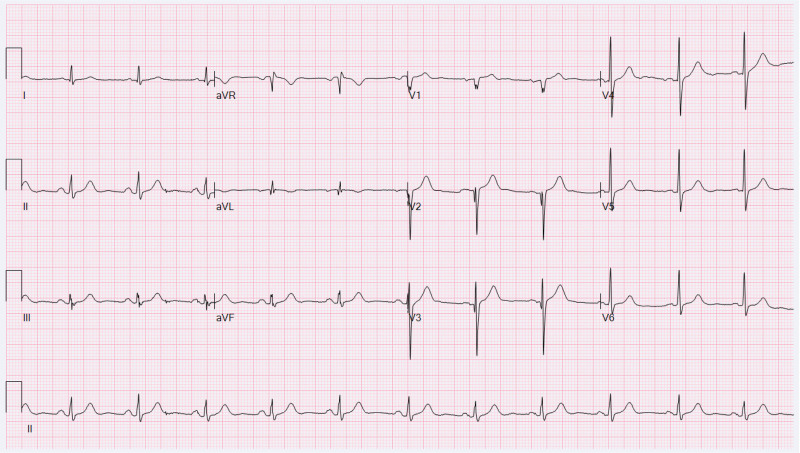
Follow-up electrocardiogram obtained 2 days after presentation.

A cardiology consultation was obtained. The ECG was interpreted as showing sinus rhythm with ST-segment elevation (0.1–0.35 mV) in leads V1–V3 and upright T waves. The absence of a notched S waves in the left precordial leads ruled out right bundle branch block. The β-angle in lead V2 was 60°, and the base of the r’-wave triangle (at a height of 5 mm) measured 340 ms (8.5 mm) (Fig. 2), consistent with a type 2 Brugada pattern. The differential diagnosis for the ST-segment elevation included ventricular aneurysm, re-occlusion of the stented artery, and acute pericarditis. Normal cardiac biomarkers and the absence of wall motion abnormalities ruled out re-occlusion and acute pericarditis. Acute pericarditis was further excluded due to the absence of diffuse ST-segment depression in lead VR. A final diagnosis of COVID-19-induced Brugada phenocopy was made. The patient was treated with oral clopidogrel at a loading dose of 300 mg, followed by a maintenance dose of 75 mg daily, oral acetaminophen (0.5 g) for fever, and oral nirmatrelvir/ritonavir (300 mg/100 mg) as antiviral therapy. After 2 days, his temperature normalized to 37.2°C, and a repeat ECG (Fig. 3) showed complete resolution of ST-segment elevation. No Brugada pattern was observed on follow-up ECGs after hospital discharge.

## 4. Discussion

This case demonstrates a transient type 2 Brugada pattern triggered by COVID-19 infection in a febrile patient with a history of myocardial infarction. The diagnosis and differentiation of Brugada patterns are key areas of study in electrophysiology. The differential diagnosis includes acute coronary syndrome, early repolarization (ER), complete right bundle branch block, arrhythmogenic right ventricular cardiomyopathy (ARVC), and normal variants in healthy athletes. Chevallier et al introduced the β-angle (≥ 58°) as a diagnostic criterion,^[2]^ and Serra et al refined this criterion in 2014 by incorporating measurement of the base duration when the height of the r’-wave triangle is 5 mm in leads V1–V2. A duration of more than 160 ms indicates a type 2 Brugada pattern.^[3]^ In this patient, the β-angle measured in lead V2 was 60°, and the base of the triangle at a height of 5 mm (x) was 340 ms (8.5 mm), consistent with a type 2 Brugada ECG pattern.

In March 2020, the World Health Organization declared COVID-19 a global pandemic, which presents with a wide range of clinical manifestations, primarily affecting the lungs but also involving the heart.^[4]^ A meta-analysis of 16 studies including 2224 patients reported a 24.4% incidence of cardiac involvement among hospitalized patients with COVID-19.^[5]^ The ECG remains a vital tool for diagnosing myocardial injury and infarction, and is frequently used to assess risk and prognosis.^[6]^

In the present case, the recurrent ST-segment elevation posed a diagnostic challenge. Fever-induced Brugada ECG patterns have been reported in asymptomatic individuals with Brugada syndrome.^[7]^ A study conducted in Thailand found that fever increased the incidence of Brugada syndrome by approximately 20-fold compared with afebrile individuals (2% vs 0.1%, *P *= .0001), suggesting that asymptomatic Brugada syndrome may be more common than previously reported.^[1]^ ER and Brugada syndrome share similar pathophysiological mechanisms. Typically, ER rarely manifests with ST-segment elevation of 0.2 mV or more in leads V1–V2. Moreover, as in this patient, the J wave tends to become more prominent during fever but disappears when body temperature normalizes, thereby allowing exclusion of ER. ARVC generally does not exhibit dynamic ST-segment changes on ECG. Instead, its diagnostic hallmarks include persistent deep T-wave inversions and the presence of epsilon waves in the terminal portion of the QRS complex. Since these findings were absent in our patient, ARVC was also ruled out.

In the process of clinical diagnosis and treatment, it is essential to evaluate the possibility of acute myocarditis. Epidemiological studies indicate that viral infections are the most common cause of acute myocarditis, with approximately 18% to 80% of patients experiencing influenza-like prodromal symptoms and respiratory or gastrointestinal issues. Additionally, the condition can occur in individuals of all ages. Approximately 62% to 96% of patients with acute myocarditis exhibit abnormal ECG findings, while 58% to 70% demonstrate ST-segment elevation resembling that of acute myocardial infarction. Cardiac magnetic resonance imaging and endomyocardial biopsy are critical diagnostic methods for confirming the condition.^[8]^ In this case, the troponin levels and myocardial enzyme profile were normal, and both the symptoms and ECG findings improved after active antiviral and symptomatic treatment. Therefore, no further examinations were conducted.

Combined with the presence of abnormal Q waves in leads V1–V3, these findings suggest a Brugada phenocopy pattern induced by COVID-19. Brugada phenocopy may exhibit ECG features resembling those of Brugada syndrome but is characterized by reversible changes when the underlying triggers are controlled.^[9]^ The resolution of the Brugada pattern after antipyretic and antiviral intervention precluded the need for coronary angiography. Nevertheless, due to the patient’s ischemic background, and despite the absence of high-risk features such as syncope or a family history of sudden death, lifelong arrhythmia surveillance is warranted.

## 5. Conclusion

In conclusion, COVID-19, particularly when associated with fever, can precipitate a Brugada phenocopy, causing increase dispersion of cardiac repolarization or cause delayed depolarization in a localized area of the right ventricular outflow tract. This case underscores the importance of careful assessment of ECG findings to formulate appropriate diagnostic and treatment strategies to avoid misdiagnosis in patients with a known history of coronary artery disease presenting with ST-segment elevation.

## 6. Limitations

This report has limitations. First, an ECG recorded in the higher intercostal spaces was not performed, which might have enhanced detection of the Brugada pattern. Second, genetic testing was not conducted, preventing a definitive distinction between congenital Brugada syndrome and Brugada phenocopy.

## Acknowledgments

We would like to thank Editage (www.editage.cn) for English language editing.

## Author contributions

**Writing – original draft:** Lijiang Ding, Huayong Jin.

**Writing – review & editing:** Huayong Jin.

## References

[1] Adler A Topaz G Heller K. Fever-induced Brugada pattern: how common is it and what does it mean? Heart Rhythm. 2013;10:1375–82. 23872691 10.1016/j.hrthm.2013.07.030 PMC3832740

[2] Chevallier S Forclaz A Tenkorang J. New electrocardiographic criteria for discriminating between Brugada types 2 and 3 patterns and incomplete right bundle branch block. J Am Coll Cardiol. 2011;58:2290–8. 22093505 10.1016/j.jacc.2011.08.039

[3] Serra G Baranchuk A Bayés-De-Luna A. New electrocardiographic criteria to differentiate the type-2 Brugada pattern from electrocardiogram of healthy athletes with r’-wave in leads V1/V2. Europace. 2014;16:1639–45. 24603955 10.1093/europace/euu025

[4] Zhang Q Zhang H Yan X. Neutrophil infiltration and myocarditis in patients with severe COVID-19: a post-mortem study. Front Cardiovasc Med. 2022;9:1026866. 36312241 10.3389/fcvm.2022.1026866 PMC9614157

[5] Zou F Qian Z Wang Y Zhao Y Bai J. Cardiac injury and COVID-19: a systematic review and meta-analysis. CJC Open. 2020;2:386–94. 32838255 10.1016/j.cjco.2020.06.010 PMC7308771

[6] Ibanez B James S Agewall S. 2017 ESC Guidelines for the management of acute myocardial infarction in patients presenting with ST-segment elevation: the task force for the management of acute myocardial infarction in patients presenting with ST-segment elevation of the European Society of Cardiology (ESC). Eur Heart J. 2018;39:119–77. 28886621 10.1093/eurheartj/ehx393

[7] Aramaki K Okumura H Shimizu M. Chest pain and ST elevation associated with fever in patients with asymptomatic Brugada syndrome. Int J Cardiol. 2005;103:338–9. 16098400 10.1016/j.ijcard.2004.06.025

[8] Ammirati E Moslehi JJ. Diagnosis and treatment of acute myocarditis. JAMA. 2023;329:1098–113. 37014337 10.1001/jama.2023.3371

[9] Adytia GJ Sutanto H. Brugada phenocopy vs. Brugada syndrome: Delineating the differences for optimal diagnosis and management. Curr Probl Cardiol. 2024;49:102566. 38599558 10.1016/j.cpcardiol.2024.102566

